# Persistent annular dermatosis: Unraveling the cause of a 2-year rash

**DOI:** 10.1016/j.jdcr.2025.06.042

**Published:** 2025-07-26

**Authors:** Ayana Crawl-Bey, Willow Pastard, Ellen N. Pritchett

**Affiliations:** a Howard University College of Medicine, Washington, District of Columbia; b Department of Dermatology, Howard University College of Medicine, Washington, District of Columbia; c Drexel University College of Medicine, Philadelphia, Pennsylvania

**Keywords:** annular lesions, annular lichen planus, chronic skin rash, dermatopathology, hepatitis C, hyperpigmentation, lichen planus, penile involvement, violaceous plaques

**Fig 1:**
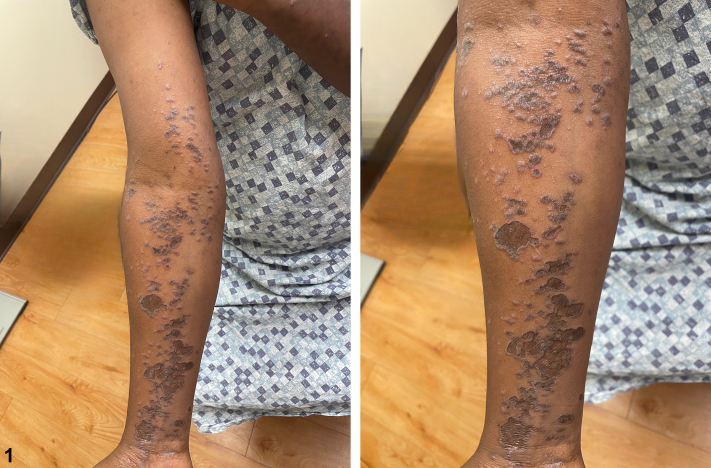

**Fig 2:**
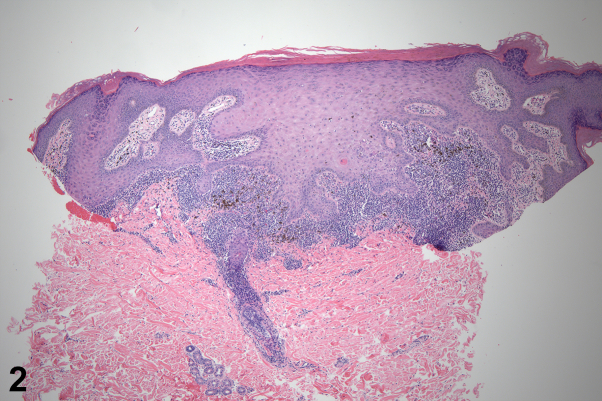

## Case description

A 34-year-old male presents for initial evaluation of a rash all over his body that first began approximately 2 years ago. After about 1 year, the rash resolved with prednisone, but new lesions appeared around 9 months ago. The lesions are asymptomatic. He also notes lesions on his penis and denies oral involvement. Examination showed diffuse violaceous annular plaques extending from the right ventral wrist to the upper arm. Similar lesions were seen on the glans penis. Punch biopsy results are shown in Fig 1. Treatment with clobetasol, hydrocortisone, and tacrolimus led to resolution of the rash. Histology of the lesion taken from biopsy of the forearm can be seen in Fig. 2.

**Question 1: What is the diagnosis?**

**A.** Annular sarcoidosis
**B.** Annular psoriasis
**C.** Annular lichen planus
**D.** Mycosis fungoides
**E.** Tinea corporis

**Answers:**

A. Annular sarcoidosis – Incorrect. Sarcoidosis can also demonstrate annular plaques with a violaceous or brownish hue. Histology reveals noncaseating granulomas, often with systemic involvement.
B. Annular psoriasis – Incorrect. Annular psoriasis typically has well-defined, raised, plaques with silvery scale and central clearing, while the most well-described variant of lichen planus appears as flat-topped, purple, polygonal papules or plaques.
C. Annular lichen planus – Correct. Annular lichen planus is a rare morphological subtype of lichen planus, characterized by ring-shaped, violaceous, or hyperpigmented plaques with slightly raised nonscaly borders.^1^ It commonly affects the male genitalia, groin, axillae, and extremities. The etiology remains unclear but is thought to involve genetic predisposition, stress, skin trauma, and infections. Lichen planus has also been associated with hepatitis C virus (HCV) infection, with studies suggesting a higher prevalence of HCV in patients with cutaneous lichen planus, particularly in endemic regions.^2^ Annular lesions are typically isolated, and histopathological examination reveals classic features of lichen planus in the active edge of the lesion.^3^ Diagnosis is primarily clinical, but histopathology can confirm the diagnosis. Treatment includes topical corticosteroids, systemic immunosuppressants, and phototherapy. Prognosis is variable, with some cases resolving spontaneously and others persisting or recurring.
D. Mycosis fungoides – Incorrect. Mycosis fungoides is the most common type of cutaneous T-cell lymphoma, which evolves slowly with patch and plaque formation with/without erythroderma, with histology showing abnormal T-cell infiltration.
E. Tinea corporis – Incorrect. Tinea corporis presents with scaly ring-shaped lesions and may be confirmed by potassium hydroxide prep showing fungal hyphae.

## Discussion

Annular lichen planus is a rare morphologic subtype of lichen planus, characterized by ring-shaped, violaceous, or hyperpigmented plaques with slightly raised, non-scaly borders.^1^ It commonly affects the male genitalia, groin, axillae, and extremities. The etiology remains unclear but is thought to involve genetic predisposition, stress, skin trauma, and infections. Lichen planus has also been associated with HCV infection, with studies suggesting a higher prevalence of HCV in patients with cutaneous lichen planus, particularly in endemic regions.^2^ Annular lesions are typically isolated, and histopathologic examination reveals classic features of lichen planus in the active edge of the lesion.^3^ Diagnosis is primarily clinical, but histopathology can confirm the diagnosis. Treatment includes topical corticosteroids, systemic immunosuppressants, and phototherapy. Prognosis is variable, with some cases resolving spontaneously and others persisting or recurring.

## Conflicts of interest

None disclosed.
